# Anti-resorptive therapy for osteoporosis and oral status of geriatric inpatients: A retrospective hospital-based study

**DOI:** 10.4317/jced.63414

**Published:** 2025-12-30

**Authors:** Elodie Scuttenaire, Adeline Braud

**Affiliations:** 1 DDS, Private Practitioner. UFR Odontologie, Université Paris Cité; 2 DDS, PhD, Professor. Service d’Odontologie, Hôpital Rothschild, APHP Sorbonne université; 3 DDS, PhD, Professor. UMR 1333 Santé Orale, Université Paris Cité

## Abstract

**Background:**

In osteoporotic patients, eliminating any sources of oral infection is recommended prior to initiating bisphosphonate (BP) therapy, as a preventive strategy against medication-related osteonecrosis of the jaw (MRONJ). Given their current state oral health status, oral disinfection before BP therapy for osteoporosis may frequently result in teeth extraction in geriatric hospitalized patients.

**Material and Methods:**

The study purpose was to determine whether the number of teeth of geriatric hospitalized patients undergoing BP therapy for osteoporosis declined during their hospital stay. A retrospective study was conducted on medical records of patients over the age of 65 who were hospitalized at the Rothschild Hospital (Assistance Publique-Hôpitaux de Paris, Sorbonne Université) and referred to the Oral Surgery Department for the detection of oral infectious foci prior to initiating antiresorptive therapy, from September 2021 to July 2022. The primary outcome measured was teeth number, recorded before the start of antiresorptive therapy and at hospital discharge. Data were analyzed by using a paired t-test, one-way ANOVA, and binary regression analysis, with statistical significance set at P&lt;0.05.

**Results:**

Among the study population (n=161 subjects, 120 women, mean age of 86.1±6.7 years), 84 subjects (52.1%) had oral infections foci and 45 subjects (27.9%) benefited from teeth extraction during the course of their hospitalization. Binary logistic regression showed that being aged 85 and above was a significant predictor of teeth extraction (p&lt;0.05), with an odds ratio of 2.63 (95% CI: 1.14-6.03). The average number of teeth significantly decreased from 16.1±9.6 to 15.4±9.7 (student t-test, p0.05, ddl= 160) during the course of the hospitalization.

**Conclusions:**

A decline in the number of teeth was observed during hospitalization. Patients aged 85 and above had a 2.6-fold higher likelihood of tooth extraction during hospitalization than younger patients.

## Introduction

As life expectancy rises, osteoporosis has emerged as a significant public health concern. In 2001, osteoporosis was estimated to cause annually around 70,000 vertebral fractures, 60,000 hip fractures, and 35,000 wrist fractures in France. The management of postmenopausal osteoporosis may involve various medications, depending on factors such as bone mineral density, age, history of fractures, and risk of falling (1): bisphosphonate (BP), osteoclast-inhibiting monoclonal antibodies (denosumab), Selective Estrogen Receptor Modulator (Raloxifene), IgG2 monoclonal antibodies (Romosozumab), recombinant parathyroid hormone analogues (teriparatide). BP are artificial counterparts of inorganic pyrophosphates, which are naturally occurring substances that contribute to the mineralization of bone tissue in human bodies (2). They have been widely used for many years as antiresorptive agents, prescribed both in the treatment of malignant pathologies (bone metastases of solid tumors, multiple myeloma) and in the prevention of osteoporotic fractures (3). In France, eight BP molecules have received marketing authorization (Etidronate, Clodronate, Tiudronate, Pamidronate, Alendronate, Risedronate, Abandronate, Zoledronate). BP can be administered either orally or intravenously (4).Taking oral BP is rather restrictive, which may reduce patient compliance. Therefore, patients with poorly managed compliance are recommended to use injectable form. In addition, the bioavailability of oral BP is very low (from 1 to 5%) compared to that of injectable BP (from 40% to 60%) (2 , 4). The main oral complication of BP is the medication-related osteonecrosis of the jaw (MRONJ), which is defined as bone exposure in the orofacial region that does not heal after eight weeks of evolution, diagnosed by a healthcare professional, in a patient who has been treated with BP and who has not had cervico-facial irradiation (5). Its incidence is between 0.8% and 12% in patients treated with injectable BP for malignant pathology while it ranges between 0.001 and 0.10% in patients treated with oral BP for benign pathologies (6). In order to prevent from MRONJ, oral examination and cavity sanitization are required before starting any BP treatment (8 , 9). According to national and international guidelines, providing dental care (cavities, endodontic treatment), reducing oral infection sources, extracting remaining roots and teeth with a poor prognosis, clearing out periodontitis or peri-implantitis sources, and modifying inappropriate removable prostheses or those causing oral lesions are all recommended (8 , 9). Thus, the oral surgeon plays a crucial role prior to starting BP therapy. They are in charge of determining whether potentially infected teeth should be retained or extracted, ensuring proper healing of extraction sites, and proposing suitable replacement tooth option. Several studies have evaluated the oral health of patients undergoing antiresorptive therapy, especially BP. Most of them focused on the profile of patients undergoing antiresorptive therapy for malignant diseases, with patients frequently aged under 65 years, and once the treatment has been initiated (10). According to Yamamoto et al. (2020), perioperative oral intervention was necessary for 10.2% of older hospitalized patients with hip fractures who required osteoporosis treatment (11). However, given that periodontitis affects up to 70% of those over 65 and dental caries impact 60.7% of older adults, the prevalence of oral contraindication to BP in geriatric hospitalized patients may be underestimated (12 , 13). Oral frailty which is defined as "the accumulation of slight declines in oral function, including tooth loss and difficulties in eating and communicating, which increases the risk of impaired oral functional capacity" (14) may reach 28% among geriatric populations (15). Considering their current state of oral health, oral disinfection prior to BP treatment in older hospitalized patients may also frequently result in teeth extraction, which may raise several ethical and clinical issues. Geriatric patients may spend a long time in hospitalization in order to gain functional abilities before going back home or moving to nursing home. When teeth are extracted during the hospital stay, prosthetic replacement of missing teeth is often delayed, occurring neither during the hospitalization period nor in the subsequent weeks after oral surgery. Thus, hospitalized patients may remain edentulous for a several weeks or months before receiving oral rehabilitation, which can contribute to increased mortality, co-morbidities such dysphagia, and overall frailty (16 , 17). For several years, the Oral Surgery Department at Rothschild Hospital has provided consultations aimed at identifying oral sources of infection prior to BP administration. Beyond assessing oral contraindications for BP therapy, the dental surgeon provides recommendations for periodontal treatment, conservative care, extraction of teeth, and oral rehabilitation throughout the patient's hospitalization. We hypothesized that the prescription of BP was associated with a reduction in the number of remaining teeth. To test this hypothesis, a retrospective study was conducted using the medical records of geriatric patients hospitalized at Rothschild Hospital (APHP Sorbonne University) who underwent oral examinations to rule out any oral infection prior to initiating antiresorptive therapy for osteoporosis. The main objective of the study was to measure the number of teeth present both prior to antiresorptive prescription and upon hospital discharge.

## Material and Methods

The protocol received the approval of the Assistance Publique-Hôpitaux de Paris Institutional Review Board (N° IRB: IORG0010044, ID 2025-02-07). In application of the French law of January 6, 1978 relative to information technology, the survey has been declared to the national data protection agency (CNIL). The survey followed the ethical principles of the Helsinki declaration and Good Clinical Practice. Anonymity of participants was respected throughout the course of the study. The reporting of data followed the STrenghtening the Reporting of Observational Studies in Epidemiology (STROBE) statement. 1. Patient selection The study population was composed of patients hospitalized at Rothschild Hospital (Assistance Publique-Hôpitaux de Paris, Sorbonne University) referred to the Oral Surgery Department for oral examination and screening for oral infectious foci prior to antiresorptive treatment, between September 1rst, 2021 to August 31, 2022. Requirements for inclusion were: age over 65, admission to a unit at Rothschild Hospital, and referred for an oral examination prior to initiating antiresorptive treatment for osteoporosis. Patients for whom the clinical examination was not performed or not available in the medical record and patients referred for other reason were excluded. From March 1 to March, 10, 2025, patients eligible for inclusion were contacted to inform them about the use of their data and to obtain their consent. This information was provided by sending an information note by post. Failure to respond within one month of the mailing was considered as consent. According to Bourgeois and Doury (18), the average number of missing teeth in the french 65-74 age group is 16.9±10.5. Consequently, the average number of teeth per individual in the French population aged over 60 is estimated to be 15.1. Given these values, and assuming a 20% reduction in the number of teeth (3.0 fewer teeth), 99 participants were needed to achieve a power (1-) of 80% with the non-parametric signed-rank test at the threshold of 5%. 2. Variables (predictor variables and outcome definition) Medical data included age (years), sex (man/women), disabilities (scores for activities of daily living (ADL) (19 , 20) and instrumental activities of daily living (IADL) (21 , 22)), number of chronic disease (23 , 24) (including cardiovascular disease, endocrine and metabolic disease, cancer, pulmonary disease, liver disease, kidney disease, mental disease and arthritis), number of medications, body mass index (BMI, kg/m2), reason and duration of hospitalization (days), time between hospital admission and oral examination (days), and living environment before and after the hospitalization. Antiresorptive therapy was also collected. The main outcome of the study was teeth number. Oral conditions were collected including the number of remaining teeth (ranging from 0 to 32), the number of occlusal functional units (OFU, ranging from 0 to 10, with one unit considered as a pair of antagonist premolars and molars), the number of decayed, missing, filled teeth (DMFT index, based on 28 teeth), the periodontal index (CPITN, ranging from 0 to 4), the existence of oral contraindication to BP, the presence of removable denture worn during meals (ranging from zero to two), the number of extracted teeth and the prosthetic oral rehabilitation managed during the hospitalization. 3. Data extraction Medical records of included patients were screened between March 1 and March, 30, 2025 and data were extracted between April 1 and June 30, 2025. After their anonymization, data were kept in an Excel file (Microsoft®). 4. Data analysis Data analysis was performed using the Statistical Package for Social Sciences (SPSS software for Windows version 11.5, SPSS®, Chicago). A descriptive analysis of the data was first carried out (means and standard deviation, distribution and frequency of responses). Based on age, two categorical variables were defined: one for individuals aged 65-84 years, and another for those over 85. Two sex group were also defined (i.e. men and women). Subjects were then categorized into three groups according to their BMI (BMI strictly below 22, BMI between 22 and 31, and BMI strictly above 31). According to the number of chronic diseases, subjects were discriminated into three groups: no disease, at least one disease, and two or more disease (25). Subjects needing help with one or more of the ADL or IADL activities were defined as ADL and IADL dependent (26). According to their living environment before and after the hospitalization, three categorical variables were considered: nursing home, individual housing, homeless. The number of teeth was categorized into three groups: equal to or &gt;20 teeth, 10-19 teeth, and &lt;10 teeth (27). Three groups of occlusal status were considered: 0-2 OFUs, 3-6 OFUs, and more than 7 OFUs (28). The null hypothesis was that the number of teeth of patient receiving BP therapy did not change during the course of the hospitalization. After verifying that the data were normally distributed, the numbers of teeth recorded at the oral examination and upon discharge from the hospital were submitted to a paired t-test. Fisher LSD significance with one-way ANOVA test and Pearson Chi-square were used to compare oral health conditions between age groups, sex groups, ADL status, IADL status, health conditions, BMI status, reason for hospitalization, and living environment before hospitalization. A binary logistic regression was performed to examine the association between teeth extraction and categorical variables. All predictors were entered simultaneously using the enter method. Significance levels were set at p0.05.

## Results

1. Study population Among the 192 patients attending the consultation during the study period, 161 fulfilled the inclusion criteria and constituted the sample study (n=120 women and n=41 men, Table 1).

Table 1

The clinical oral features of the study population are detailed in Table 1. The mean age of the subjects was 86.1±6.7 years (the youngest patient was 67 and the oldest 99) with 104 participants aged 85 years or older. The average BMI was 23.6±4.7 kg/m2, with 65 subjects having a BMI strictly below 22. The BMI status significantly varied according to sex (Chi2=6.189, p0.05) and age (Chi2=6.186, p0.05). On average, each patient had 2.9±1.5 disease (ranging from 0 to 7 chronic disease, Table 1). The distribution of disease did not vary according to sex (Chi2=0.045, p=ns) or according to age group (Chi2=0.297, p=ns). Cardiovascular disease affected 112 subjects, mental disorders 109 subjects (among them, 66 medical records mentioned neurocognitive disorders), metabolic and endocrine disorders 72 subjects, arthritis 30 subjects, kidney disease 26 subjects, and pulmonary disease 13 subjects. In addiction sensory disease including ocular pathologies affected 28 subjects with cataract affecting 10 subjects, age-related macular degeneration 6 patients and blindness 1 patients. Among the study population, 159 subjects had medications. On average, subjects took 8.2 ± 3.7 medications. Among them, 73 subjects were taking anticoagulants, 56 subjects benzodiazepines, 52 subjects antidepressants, 10 subjects hypnotics, 5 subjects anti-psychotics, and 4 subjects benserazide/levodopa. Among the study population, 82 subjects were ADL dependent and 112 subjects IADL dependent. The distribution of the ADL and IADL dependency did not vary according to sex (respectively Chi2=0.134, p=ns and Chi2=0.010, p=ns) or according to age group (Chi2=1.495, p=ns and Chi2=1.273, p=ns) The main reason for hospitalization was bone fracture, which concerned 116 subjects. Other reasons included fall without fracture (12 subjects), altered general conditions (3 subjects) and other pathologies (30 subjects) including ulcers and bedsores, osteonecrosis of the head of the femur, dissecting hematoma, hallux amputation and radiculalgia, pyelonephritis, urinary tract infection, intestinal obstruction or recto sigmoiditis, rheumatoid arthritis, febrile dyspnea, and cardiac decompensation. The average duration of hospital stay was 80±65 days (ranging from 10 days to 559 days). Before the hospitalization, 150 subjects were living at home, 10 subjects lived in a nursing home and one patient was homeless. Upon leaving the hospital, 91 subjects went on to live in a nursing home, while 60 returned to individual housing. Forty-nine subjects who had been living in a single-family home prior to hospitalization moved into a nursing home upon discharge form hospital. Six subjects died during their hospitalization (2 men and 4 women). 2. Antiresorptive therapy for osteoporosis Among the study sample, 129 subjects were referred with mention of a BP prescription. For the remaining 32 patients, the referral letter did not specify either the medication prescribed or the admission procedures. During the course of the hospitalization, 82 subjects received antiresorptive therapy, with up to four different types of molecules: BP (zoledronic acid, n=77), parathyroid hormone analog (teriparatide, n=2), anti-RANK ligand (denosumab, n=2), and cinacalcet hydrochloride (mimpara, n=1). Seventy-nine subjects did not receive antiresorptive treatment during hospitalization, either due to a re-evaluation of the indication for BP, which was subsequently withdrawn by the medical team (n = 41), or because treatment was planned post-discharge - although in such cases, it remains uncertain whether the therapy was ultimately initiated (n = 38 patients). As the number of patients receiving non-BP molecules (e.i. mimpara and teriparatide) was small, we categorized patients into two groups based on antiresorptive therapy for further analyses: those who received BP or denosumab during their hospital stay (n=79), and those who received non-BP medications or no therapy at all during hospitalization (n=82). 3. Oral status The average time between the hospital admission and the oral examination was 35±56 days (ranging from 2 days to 539 days). At the oral examination, the mean±SD teeth number was 16.1±9.6. Fifteen subjects were fully dentate (28 to 32 teeth). At the time of the consultation, there were 41, 49, and 71 patients with strictly fewer than 10 teeth, between 10 and 19 teeth, and more than 20 teeth, respectively (90 patients had fewer than 20 teeth) Twenty-three subjects were completely edentulous. Among them, 6 subjects had teeth not replaced, 2 subjects had only upper denture, 15 subjects had complete upper and lower dentures. Forty-two subjects were partially dentate. Among them, 16 subjects had partial dentures. The average DMFT score was 19.7±7.0 (ranging from 2 to 28). DMFT scores varied according to age (F(1, 160)=5.9732, p&lt;0.05). The CPITN score was mentioned in 118 medical records and had a mean±SD estimated to 2.5±0.7 (ranging from 0 to 4). CPITN scores did not vary according to age (F(1, 117)=0.362, p=0.548). OFU number was filled in 145 medical records and had a mean±SD equal to 5.2±3.1 (ranging for 0 to 10, with 39 subjects having 0-2 OFUs, 40 having 3-6 OFUs, and 66 having at least 7 OFUs). Among the study population, 84 subjects had oral infection foci. The distribution of oral infection foci significantly varied according to age (Chi2=46.8215, p&lt;0.05) but did not vary according to sex (Chi2=2.785, p=0.095), general conditions (Chi2= 0.669, p=0.715), ADL status (Chi2=0.392, p=0.531), IADL status (Chi2=0.432, p=0.510), reason for hospitalization (Chi2=2.475, p=540), BMI status (Chi2=3.546, p=0.576), and lifestyle before hospitalization (Chi2=0.937, p=0.625). Of the 84 subjects with oral infection foci, 45 had teeth removed during their hospitalization, 26 refused teeth removal, 11 had oral surgery canceled following reassessment of anti-resorptive treatment, and 2 were did not attend their surgical appointment. Teeth extraction concerned 18 subjects receiving either zoledronic acid or denosumab. Univariate analysis showed that teeth extraction significantly varied with age (Chi2=5.999, p0.05) but did not vary according to sex (Chi2=0.838, p=0.360), health conditions (Chi2=11.807, p=0.107), ADL status (Chi2=0.01, p=0.970), IADL status (Chi2=1.388, p=0.238), reason for hospitalization (Chi2=4.347, p=0.629), BMI status (Chi2=3.546, p=0.169), lifestyle before hospitalization (Chi2=2.861, p=0.239) and antiresorptive therapy (Chi2=2.238, p=0.132). A binary logistic regression showed that being aged 85 and above was a significant predictor of teeth extraction (p = 0.023), with an odds ratio of 2.63 (95% CI: 1.14-6.03, Table 2).

Table 2

All other categorical variables were not statistically significant, although a few showed borderline trends (i.e., BMI above 31, OR = 0.278, p = 0.083; IADL status: OR = 2.882, p = 0.102). Over the study population, the average number of teeth fell from 16.1±9.6 to 15.4±9.7 over the course of the hospitalization (student's t-test, p 0.05, ddl= 160). The mean±SD number of removed teeth was 2.6±2.8. The number of teeth significantly decreased for subjects who benefited from zoledronic acid, and those who had no antiresorptive therapy during the hospital stay (Table 3).

Table 3

One-way ANOVA showed that the number of extracted teeth significantly varied according to reason for hospitalization (F(5, 156)=3.617, p&lt;0.05) but did not vary according to age (F(5, 156)=0.312, p=0.577), sex (F(5, 156)=0.650, p=0.421), ADL status (F(5, 156)=0.006, p=0.938), IADL status (F(5, 156)=0.106, p=0.744), general health conditions (F(5, 156)=0.952, p=0.388), BMI status (F(5, 156)=0.567, p=0.568), lifestyle before hospitalization (F(5, 156)=0.377, p=0.686), and osteoporosis therapy (F(5, 156)=2.173, p=0.142). The distribution of the dental status significantly varied alongside the hospitalization (Chi2=255.009, p0.05). Upon discharge of the hospital, 47 and 49 subjects had respectively 0-9 teeth and 10-19 teeth (i.e.96 patients (59.6%) had strictly less than 20 teeth). The number of OFU did not vary alongside the hospitalization (mean±SD at the time of the oral examination and upon discharge from the hospital were respectively 5.1±3.1 and 5.1±3.1, p=ns, ddl=144). At the time of the dental examination, 39 subjects had 0-2 OFUs and 40 had 3-6 OFUs. Only one subject who benefited from teeth removal had partial denture fabrication during their hospitalization. Upon discharge from the hospital, 38 and 44 subjects had 0-2 and 3-6 OFUs, respectively (Chi2=252.717, p&lt;0.05).

## Discussion

Does the number of teeth decrease when BP are prescribed for osteoporosis in hospitalized geriatric patients? To address this question, we performed a retrospective analysis of medical data from hospitalized older adultsreferred for oral examination before initiating antiresorptive therapy. The data collected provided insight into the clinical characteristics of hospitalized patients who underwent an oral examination prior to the initiation of antiresorptive therapy at Rothschild Hospital. With an average age of about 86, primarily made up of women, suffering from an average of 4.6 ± 1.7 disease and consuming daily 5.3 ± 2.2 drugs, the study population well represented French geriatric populations (29 , 30). Notably, half of the patients needed assistance with activities of daily living, including oral hygiene, while more than 2/3 needed help with instrumental activities such as organizing of regular professional oral follow-up. This finding is all the more astonishing given that the majority of included patients lived independently prior to hospitalization (only 8% of the study population lived in an institution), which illustrates how older people remain at home despite a decline in their autonomy for daily activities. The hospitalization seems in fact to be a breaking point for these patients, since 37.1% of those who lived in individual housing prior to hospitalization moved to a nursing home upon discharge. As previously observed in hospitalized geriatric populations (31), the prevalence of malnutrition reached 40% within the study sample. In addition, 72% of included patients were hospitalized following bone fracture, and may thus suffer from restricted mobility. According to Fried (32), frailty phenotype includes clinical criteria such as weight loss, exhaustion, weakness, slow walking speed, and low physical activity. In geriatric populations, bone fractures result mostly from bone fragility and are a primary cause of functional disability and a systemic decline (33 - 35). Patient frailty was not documented in the medical records, but based on general indicators, it is likely that some patients were frail. BP have been the widely used in osteoporosis older patients for more than two decades. The prescription of BP is actually commonly practiced in the geriatric units of Rothschild Hospital, as 80% of the patients referred for an oral check-up were initially identified as candidates for BP therapy. Other antiresorptive therapies are now available for osteoporosis conditions for few years including denosumab, a monoclonal antibody against RANKL that potently inhibits osteoclast development and activity, teriparatide and abaloparatide which both target the parathyroid hormone-1 receptor, and romosozumab which is an anti-sclerostin monoclonal antibody that stimulates bone formation and inhibits resorption. Teriparatide and abaloparatide do not appear to have significant adverse effects on bone healing. Major risk of BP therapy (and denosumab) is MRONJ. Local factors of MRONJ related to BP therapy have been identified including invasive oral procedures, periodontal disease, and poor oral hygiene (36). In addition, chronic oral infections (such as failed endodontic treatments, untreated apical periodontitis, severe periodontal disease, and pericoronitis) (36) may function as independent risk factors of MRONJ, by sustaining a state of ongoing local inflammation and compromising bone integrity (37). In patients receiving oral BP for osteoporosis, the incidence of MRONJ is estimated at less than 0.1% (38 , 39). MRONJ treatment includes non-surgical interventions (i.e. administration of topical antimicrobial mouth rinses and antibiotics in order to improve the stage of disease and healing) and operative surgical management methods (i.e. marginal resection of the bone mandible or maxilla) (40), which may result in serious functional issues. Maintaining good oral hygiene and regular oral follow-up are baseline essential preventive measures of MRONJ. Prior to initiating BP therapy, oral examinations and treatments are also advised in order to eliminate local factors like advanced periodontal disease, deep caries with pulpal involvement, or periapical lesions, as well as to lower the risk of invasive procedures like tooth extraction following BP introduction (8 , 9). For the past ten years, oral check-ups have been a crucial component of geriatric care at Rothschild Hospital. The service is used in the event of oral emergencies during hospitalization, denture incident, or to detect infectious outbreaks. The clinical oral examination currently revealed poor oral health indicators including a low average number of teeth, and high DMFT and CPITN scores. The maintenance of satisfactory oral health by the patient and/or their caregivers may be compromised by the patient's dependency on instrumental activities of daily living. Some patients may also face several obstacles to receiving oral professional care, including diminished autonomy, social isolation, and physical challenges in moving about, which can lead to impaired oral conditions. Maintaining good oral health is moreover challenging for hospitalized patients. During hospitalization, between-meal consumption of sweet foods and drinks frequently rises, although the frequency of tooth brushing decrease (41). In addition, we observed that 49% of patients had less than 7 OFUs, and may thus suffer from oral frailty. Previous research have revealed a significant relationship between oral frailty and increased risk of malnutrition, physical frailty, sarcopenia, long-term care needs, and premature mortality (42 , 43). The consequences of declining oral health are amplified in the older population, especially during hospitalization. In older hospitalized patients, poor oral health status at admission is associated with longer hospital stays and significantly increases the risk of mortality (44). Clinical examination finally demonstrated that 52.1% of patients exhibited an oral contraindication to BP therapy. This rate seems particularly high compared to that previously observed by Yamamoto et al., (11) showing that 10.2% older hospitalized patients with hip fracture required perioperative oral intervention to treat oral hygiene and infectious lesions before BP. However, this rate appears to be closer to what was observed by Inoue et al., (2023), who reported that 86.8% of hospitalized older patients with hip fracture could not be prescribed postoperative BP at discharge due to oral hygiene problems, lack of regular dental consultations, renal dysfunction, poor cognitive and swallowing functions, and medication side effects (45). Anyway, impaired oral health at admission may be a contributing factor to the increased occurrence of BP contraindications in this cohort. Among the study population, 27.9% of patients had teeth extracted following the oral examination. While this rate may seem low, it is likely due to patients refusing surgery during their hospital stay, those being discharged before the planned procedure, or when the surgery was canceled by either the medical team or the patient. The analysis indicates that age was a significant factor of tooth extraction, with individuals aged 85 and over being 2.6 times more likely to have teeth removed than their younger counterparts. This finding prompts important questions about medical decision-making and the care delivered to the oldest patients, who frequently face vulnerabilities related to loss of autonomy. Could this result stem from an ageist attitude towards the oldest patients? Or could it simply result from the poor oral health status of the patients? This possibility seems likely because individuals aged 85 and older had a higher DMFT index (mean ± SD = 20.7 ± 6.6) than those aged 65 to 84 (mean ± SD = 17.9 ± 7.4). Statistical analyses also revealed that the teeth number of the sample study fell from 16.1±9.6 to 15.4±9.7 over the course of the hospitalization, which suggests that antiresorptive therapy prescription may negatively impact the dental status of geriatric inpatients. One could argue that that this reduction holds limited clinical relevance, given that the average loss was estimated at less than one tooth. However, the routine extraction of teeth following dental consultations affects patients with pre-existing poor dental status (i.e., teeth number lower than 20), and compromised occlusal status (i.e., OFU number lower than 2) which further reduces their masticatory capacity (46). It is acknowledged that the risk of malnutrition is higher in older adults who lack functional dentition (47). Given the high proportion of malnourished patients in the study, it can be inferred that the deterioration of masticatory function in patients with poorly or non-functional dentition could further exacerbate their clinical condition. As expected, patients who initiated BP therapy during their hospitalization demonstrated a significant decrease in teeth number. However, statistical analyses did not reveal that BP prescription was a significant factor of teeth extraction among the sample study. What surprised the team was that patients who did not receive any resorptive treatment during their hospitalization also exhibited a decline in their dental status. This means that these patients benefited from tooth extraction during their hospitalization but are unlikely to benefit from BP therapy after discharge. This raises questions about the intra-hospital care pathway and communication between healthcare professionals regarding molecules and treatment schedules. Illness, injury, and surgical interventions (including tooth extraction) can be significant sources of stress for older people (48). Physical resilience (i.e., ability to withstand clinical stressors and quickly recover upon a baseline functional level) is closely related to intrinsic capacity (i.e., physical and mental conditions) (49). When ageing, intrinsic capacity decreases while frailty increases (50). Tooth extraction may adversely impact both the physical and mental health of older adults, with potentially more pronounced effects in individuals exhibiting reduced physiological resilience. Given the average length of hospitalization, estimated to exceed two months, the clinical impact of teeth removal could be even more detrimental for patients. One patient only had prosthetic care during their hospitalization. For other patients, teeth removed were not systematically replaced during the weeks following the oral surgery. Teeth loss may be responsible of a change in eating habits, such as avoiding specific foods like meat, fruits or vegetables. It is necessary to reconsider the oral healthcare pathway for patients admitted in this situation, considering the duration of hospitalization and the patient's post-discharge prospects. To provide a cohesive care pathway, scheduling oral rehabilitation and surgery in collaboration with the geriatric team and caregivers is a must for care. Ethical considerations may also be raised about patients suffering from neurocognitive disorders. Sixty-six medical records presently mentioned cognitive decline without indicating the level of memory impairment and the onset of behavioral disorders. For these patients, the number of teeth fell from 16.1±9.5 to 15.6±9.6 (p0.05, t-test). The clinical management of patients suffering from neurocognitive decline is often complex, due to their legal status (legal protection proceedings in progress or effective), the difficulty in obtaining consent for treatment, a lack of compliance or even opposition to treatment. We may thus presume that prosthetic rehabilitation will never be proposed to these patients. For these patients, it seems essential to consider the benefits and consequences of oral care, in collaboration with the geriatric team, with the goal of buccal comfort and care security. Several aspects of the research constitute limitations in the interpretation of the results. Based on the retrospective analysis of medical records, the data collection may suffer from the lack of information and the absence of clinical follow up of patients. The majority of patients did not receive follow-up care from the dental department after being discharged from the hospital. For the other, only one medical record presently mentioned the occurrence of MRONJ between the discharge from hospital (2021-2022) and the data collection (2025). Furthermore, the results' generalizability may be limited by selection bias introduced by the single-center retrospective methodology. The second bias can arise from the manner that physicians who are in charge of hospitalization prescribed medications for osteoporosis and adjusted the prescription during the hospital stay. Lastly, the medical records of 38 patients did not mention any antiresorptive therapy following the dental examination. It is unclear whether these patients received treatment or discontinued it after they were released from the hospital.

## Conclusions

Conclusions Clinical examination revealed poor oral health prior to antiresorptive therapy, and identified oral infection foci in 52.1% of hospitalized geriatric patients. Although only 27.9% of patients benefited from teeth extraction during their hospitalization, the number of teeth was significantly reduced during the course of the hospitalization. Almost no patients received oral prosthetic rehabilitation during their hospitalization. Beyond national and international recommendations on BP therapy for osteoporosis, tooth loss during hospitalization can have dramatic consequences on the health of frail older patients.

## Figures and Tables

**Table 1:** Mean characteristics of the study sample.

	Women (n=120)	Men (n=41)	P Value*
	Mean±SD		
Age (years)	86.4±6.9	85.1±6.3	ns
BMI (kg/m2)	23.6±4.9	23.3±3.8	ns
Disease number	2.9±1.5	2.7±1,5	ns
Medication number	8.0±3.7	8.7±3.7	ns
Teeth number	16.0±9.9	16.0±8.6	ns
DMFT score	19.9± 7.2	19.4± 6.6	ns
CPITN score	2.5±0.8	2.5±0.7	ns
OFU number	5.3±3.0	4.5±3.3	ns

ns: not significant. Note: Statistical significance is set at P< .05.*Analysis of variance

**Table 2:** Logistic regression model for the association between teeth extraction and health variables. (CI = confidence interval; OR = odds ratio).

	B	SD	Chi2	p	Exp (B)	CI 95%	
Age 85 and above	0.969	0.422	5.266	0.022	2.636	1.152	6.031
Women	0.350	0.477	0.539	0.463	1.419	0.558	3.611
BMI below 22	-0.286	0.442	0.419	0.517	0.751	0.316	1.787
BMI above 31	-1.281	0.740	3.000	0.083	0.278	0.065	1.184
ADL dependent	-0.517	0.536	0.929	0.335	0.596	0.208	1.706
IADL dependent	1.059	0.647	2.679	0.102	2.882	0.811	10.241
Bone fracture	0.486	0.437	1.238	0.266	1.626	0.691	3.826
Life at home	0.230	0.877	0.068	0.794	1.258	0.225	7.025
BP therapy	0.778	1.313	0.352	0.553	2.178	0.166	28.537
One disease	1.011	1.314	0.592	0.442	2.749	0.209	36.139
At least 2 disease	-0.625	0.579	1.162	0.281	0.535	0.172	1.667

2

**Table 3:** Number of teeth before the oral examination and upon discharge from hospitalization.

		teeth number before oral check-up	teeth number upon discharge from hospitalization	p
Follow-up	n	Mean±SD		
zoledronic acid	102	15.2±9.9	14.8±9.9	<0.05
denosumab	2	13±18.4	11±15.6	ns
teriparatide	2	23±5.6	23±5.6	ns
mimpara	1	14	14	ns
BP therapy cancelled	41	16.9±9.2	16.5±9.1	<0.05
antiresorptive therapy scheduled after hospital discharge	38	16.8±8.5	15.1±9.3	<0.05

3

## Data Availability

The datasets used and/or analyzed during the current study are available from the corresponding author.

## References

[1] Briot K, Roux C, Thomas T, Blain H, Buchon D, Chapurlat R (2018). 2018 update of French recommendations on the management of postmenopausal osteoporosis. Joint Bone Spine.

[2] Drake MT, Clarke BL, Khosla S (2008). Bisphosphonates: mechanism of action and role in clinical practice. Mayo Clin Proc.

[3] Papapoulos SE, Makras P (2025). Bisphosphonates in the Management of Patients with Postmenopausal Osteoporosis; Back to the Future. Pharmaceuticals (Basel).

[4] Kim KJ (2025). From Bone Health to Lifespan: Pleiotropic Effects of Antiresorptive Agents. Endocrinol Metab (Seoul).

[5] Khosla S, Burr D, Cauley J, Dempster DW, Ebeling PR, Felsenberg D (2007). Bisphosphonate associated osteonecrosis of the jaw: report of a task force of the American Society for Bone and Mineral Research. J Bone Miner Res.

[6] Kostares E, Kostare G, Kostares M, Pitsigavdaki F, Perisanidis C, Kantzanou M (2025). Prevalence of Osteonecrosis of the Jaw Following Tooth Extraction in Patients with Osteoporosis: A Systematic Review and Meta-Analysis. J Clin Med.

[7] Baghalipour N, Moztarzadeh O, Samar W, Gencur J, Volf V, Hauer L (2025). Comprehensive Review of Prevention and Management Strategies for Medication-related Osteonecrosis of the Jaw (MRONJ). Oral Health Prev Dent.

[8] Tilotta F, Folliguet M, Radoï L (2023). Preserving the oral health of patients on antiresorptive drugs. Rev Prat.

[9] Ruggiero SL, Dodson TB, Aghaloo T, Carlson ER, Ward BB, Kademani D (2022). American Association of Oral and Maxillofacial Surgeons’ Position Paper on Medication-Related Osteonecrosis of the Jaws-2022 Update. J Oral Maxillofac Surg.

[10] Schwech N, Nilsson J, Gabre P (2023). Incidence and risk factors for medication-related osteonecrosis after tooth extraction in cancer patients-A systematic review. Clin Exp Dent Res.

[11] Yamamoto N, Sukegawa S, Sukegawa-Takahashi Y, Honda T, Furuki Y, Kawasaki K, Ozaki T (2020). Relationship between oral condition and risk factors for jaw osteonecrosis in patients with hip fractures. J Med Invest.

[12] Almasvandi Y, Ziaei N, Kazeminia M, Abbasi P (2025). Global prevalence of dental caries in the older people, 1991 to 2024: a systematic review and meta-analysis. Saudi Dent J.

[13] Huang X, Kang L, Bi J (2025). Epidemiology of oral health in older adults aged 65 or over: prevalence, risk factors and prevention. Aging Clin Exp Res.

[14] Tanaka T, Hirano H, Ikebe K, Ueda T, Iwasaki M, Minakuchi S, Arai H, Akishita M, Kozaki K, Iijima K (2024). Consensus statement on “Oral frailty” from the Japan Geriatrics Society, the Japanese Society of Gerodontology, and the Japanese Association on Sarcopenia and Frailty. Geriatr Gerontol Int.

[15] Zhou Y, Zhou L, Zhang W, Chen Y, She K, Zhang H, Gao Y, Yin X (2024). Prevalence and influencing factors of oral frailty in older adults: a systematic review and meta-analysis. Front Public Health.

[16] Chauhan N, Paul S, Purohit BM, Duggal R, Priya H, S S (2025). Nutrition and oral health-related quality of life (OHRQoL) in older adults: a systematic review and meta-analysis. Evid Based Dent.

[17] Shyu SW, Lin CF, Yang SH, Chu WM, Hsu CY, Lin SY (2024). Association of oral health with geriatric syndromes and clinical outcomes in hospitalized older adults. J Nutr Health Aging.

[18] Bourgeois DM, Doury J (1999). Periodontal conditions in 65-74-year-old adults in France, 1995. Int Dent J.

[19] Spector WD, Katz S, Murphy JB, Fulton JP (1987). The hierarchical relationship between activities of daily living and instrumental activities of daily living. J Chronic Dis.

[20] Candela F, Zucchetti G, Ortega E, Rabaglietti E, Magistro D (2015). Preventing loss of basic activities of daily living and instrumental activities of daily living in elderly: identification of individual risk factors in a holistic perspective. Holist Nurs Pract.

[21] Lawton MP, Brody EM (1969). Assessment of older people: self-maintaining and instrumental activities of daily living. Gerontologist.

[22] Lawton MP (1971). The functional assessment of elderly people. J Am Geriatr Soc.

[23] Chen Y, Hicks A, While AE (2014). Quality of life and related factors: a questionnaire survey of older people living alone in mainland China. Qual Life Res.

[24] Storeng SH, Sund ER, Krokstad S (2018). Factors associated with basic and instrumental activities of daily living in elderly participants of a population-based survey: the Nord-Trøndelag health study, Norway. BMJ Open.

[25] Jang JH, Kim JR, Kim JH (2021). Association between denture use, chewing ability, and all-cause mortality in middle-aged and older adults who exercised regularly in Korea. Sci. Rep.

[26] van der Vorst A, Op Het Veld LPM, De Witte N, Schols JMGA, Kempen GIJM, Zijlstra GAR (2018). The impact of multidimensional frailty on dependency in activities of daily living and the moderating effects of protective factors. Arch Gerontol Geriatr.

[27] Jun NR, Kim JH, Park JT, Jang JH (2022). Association of number of teeth with ADL/IADL in Korean middle-aged and older adults: an analysis of the 7th Korean longitudinal study of aging. Int. J. Environ. Res. Public Health.

[28] Hildebrandt GH, Dominguez BL, Schork MA, Loesche WJ (1997). Functional units, chewing, swallowing, and food avoidance among the elderly. J Prosthet Dent.

[29] Boucaud-Maitre D, Meillon C, Letenneur L, Villeneuve R, Dartigues JF, Amieva H (2023). Health trajectories of elderly living in French senior housing: a longitudinal perspective. Sci Rep.

[30] Van Dendaele E, Pothier K, Bailly N (2024). Profiles of well-being in French older adults and associations with successful aging and personality: findings from the SHARE project. Aging Clin Exp Res.

[31] Raynaud-Simon A, Revel-Delhom C, Hébuterne X (2011). Clinical practice guidelines from the French Health High Authority: nutritional support strategy in protein-energy malnutrition in the elderly. Clin Nutr.

[32] Fried LP, Tangen CM, Walston J, Newman AB, Hirsch C, Gottdiener J (2001). Cardiovascular Health Study Collaborative Research Group. Frailty in older adults: evidence for a phenotype. J Gerontol A Biol Sci Med Sci.

[33] Andaloro S, Cacciatore S, Risoli A, Comodo RM, Brancaccio V, Calvani R (2025). Hip Fracture as a Systemic Disease in Older Adults: A Narrative Review on Multisystem Implications and Management. Med Sci (Basel).

[34] Bajeux E, Corvol A, Somme D (2021). Integrated Care for Older People in France in 2020: Findings, Challenges, and Prospects. Int J Integr Care.

[35] Dyer SM, Crotty M, Fairhall N, Magaziner J, Beaupre LA, Cameron ID (2016). A critical review of the long-term disability outcomes following hip fracture. BMC Geriatr.

[36] Rosella D, Papi P, Giardino R, Cicalini E, Piccoli L, Pompa G (2016). Medication-related osteonecrosis of the jaw: Clinical and practical guidelines. J. Int. Soc. Prev. Community Dent.

[37] Stelea CG, Bologa E, Boișteanu O, Platon AL, Gelețu GL (2025). Bisphosphonate-Related Osteonecrosis of the Jaw: A 10-Year Analysis of Risk Factors and Clinical Outcomes. J Clin Med.

[38] Tempesta A, Capodiferro S, Di Nanna S, D’Agostino S, Dolci M, Scarano A (2023). Medication-related osteonecrosis of the jaw triggered by endodontic failure in oncologic patients. Oral Dis.

[39] McGowan K, McGowan T, Ivanovski S (2018). Risk factors for medication-related osteonecrosis of the jaws: A systematic review. Oral Dis.

[40] Boston B, Ipe D, Capitanescu B, Gresita A, Hamlet S, Love R (2023). Medication-related osteonecrosis of the jaw: A disease of significant importance for older patients. J Am Geriatr Soc.

[41] Varoni EM, Lombardi N, Villa G, Pispero A, Sardella A, Lodi G (2021). Conservative Management of Medication-Related Osteonecrosis of the Jaws (MRONJ): A Retrospective Cohort Study. Antibiotics.

[42] Zhu SR, Wei LY, Jia K, Xie YX, Tan ZK, Mo ST (2024). Prevalence and unfavourable outcome of oral frailty in older adult: a systematic review and meta-analysis. Front Public Health.

[43] Weiss S, Tinsky N, Oren L, Chodick G, Spierer S, Yarom N (2024). Effect of prolonged hospitalization on the maintenance of oral health: A self-report survey. Int J Dent Hyg.

[44] Yamaguchi K, Miyagami T, Imada R, Kushiro S, Yanagida R, Morikawa T (2024). Effect of poor oral health status at hospital admission on in-hospital outcomes of older patients with aspiration pneumonia. Eur Geriatr Med.

[45] Inoue H, Oyama R, Nakamura K, Inokuchi A, Hamada T, Izumi T (2023). Bisphosphonates Prescription for Patients With Hip Fractures Based on Evaluation by a Dentist. Cureus.

[46] Kaurani P, Kakodkar P, Bhowmick A, Samra RK, Bansal V (2024). Association of tooth loss and nutritional status in adults: an overview of systematic reviews. BMC Oral Health.

[47] Zelig R, Goldstein S, Touger-Decker R, Firestone E, Golden A, Johnson Z (2022). Tooth Loss and Nutritional Status in Older Adults: A Systematic Review and Meta-analysis. JDR Clin Trans Res.

[48] Colon-Emeric C, Schmader K, Cohen HJ, Morey M, Whitson H (2023). Ageing and physical resilience after health stressors. Stress Health.

[49] Chhetri JK, Xue QL, Ma L, Chan P, Varadhan R (2021). Intrinsic Capacity as a Determinant of Physical Resilience in Older Adults. J Nutr Health Aging.

[50] Ma L, Chhetri JK, Zhang Y, Liu P, Chen Y, Li Y, Chan P (2020). Integrated Care for Older People Screening Tool for Measuring Intrinsic Capacity: Preliminary Findings From ICOPE Pilot in China. Front Med (Lausanne).

